# Dislocation of a middle lobe torsion-preventing bridging structure with an absorptive sheet and fibrin glue: a case report

**DOI:** 10.1186/s40792-022-01562-y

**Published:** 2022-11-08

**Authors:** Hiroki Matsumiya, Koji Kuroda, Masataka Mori, Masatoshi Kanayama, Akihiro Taira, Shinji Shinohara, Masaru Takenaka, Fumihiro Tanaka

**Affiliations:** Second Department of Surgery, School of Medicine, University of Occupational and Environmental Health, Yahatanishi, Kitakyushu, 807-8555 Japan

**Keywords:** Middle lobe rotation, Middle lobe torsion, Postoperative complications

## Abstract

**Background:**

Middle lobe torsion is a rare complication of right upper lobectomy. Middle lobe torsion can be critical; thus, various preventive measures are used.

**Case presentation:**

A 77-year-old man underwent thoracoscopic right upper lobectomy with partial middle resection and S6 segmentectomy for right upper lobe lung cancer located at the confluence of the three lobes and lower lobe lung cancer. Inversion of the middle lobe was observed during lung expansion before chest closure. A bridging structure with an absorptive sheet and fibrin glue was placed in the basal section of the middle lobe under lung expansion to prevent torsion. On postoperative day 1, the patient was tachycardic and was found to have decreased lung field permeability. The patient underwent emergency surgery for suspected middle lobe torsion. Dislocation of the bridging structure between the basal segments of the middle lobe was confirmed, and the middle lobe was deviated cephalad. In addition, pulmonary congestion in S4 due to pressure stenosis of V4 caused by the deviation of the middle lobe was observed, and middle lobe resection was performed. The postoperative course was uneventful.

**Conclusions:**

This case suggested that the reinforcement method with an absorptive sheet and fibrin glue lacked sufficient strength to prevent middle lobe torsion. Stronger fixation should be considered if the middle lobe rotation is thought to be sufficiently strong when the lung is reinflated before chest closure.

**Supplementary Information:**

The online version contains supplementary material available at 10.1186/s40792-022-01562-y.

## Background

Middle lobe torsion following right upper lobectomy is the most frequent type of lobe torsion [1]. Torsion is defined as a parenchymal rotation on the bronchovascular pedicle accompanied by vascular compromise and obstruction of the associated airway and may require emergent surgery [2]. Various methods to prevent torsion have been reported [3], with the simple absorptive sheet and fibrin glue method preferred in our hospital [4]. However, we experienced a case of torsion despite these measures, which had not been a problem in the past, and report this case together with the operative findings.

## Case presentation

**Fig. 1:**
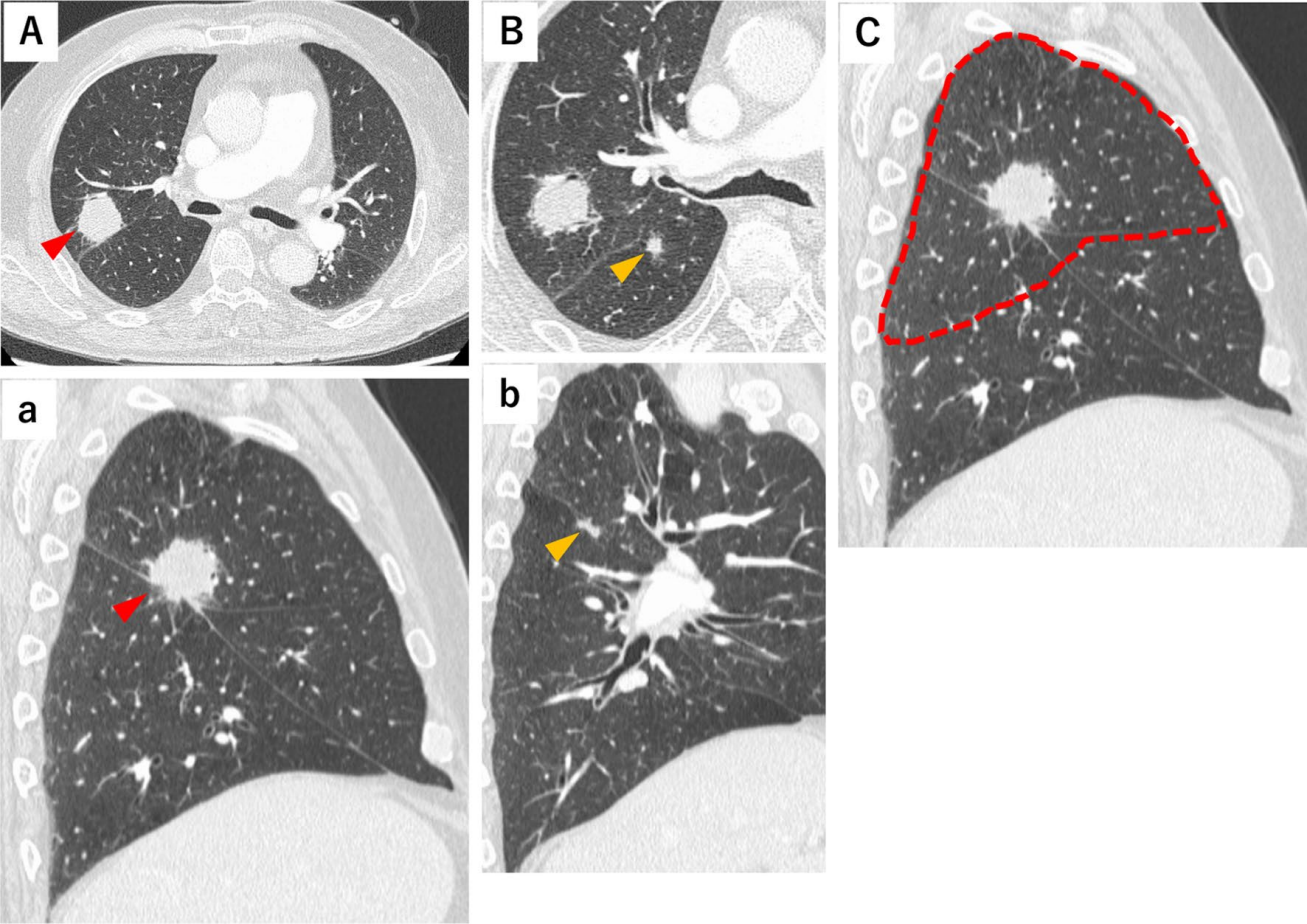
**A** Right upper lobe lung cancer (adenocarcinoma, pT2a (32 mm), N0M0, Stage IB). (a) Tumor is located near the trilobar confluence. Good lobulation of the middle and lower lobes. **B** (b) Right lower lobe lung cancer (squamous cell carcinoma, pT1a (7 mm), N0M0, Stage IA1). **C** Extent of the resected lung in the sagittal section

**Fig. 2:**
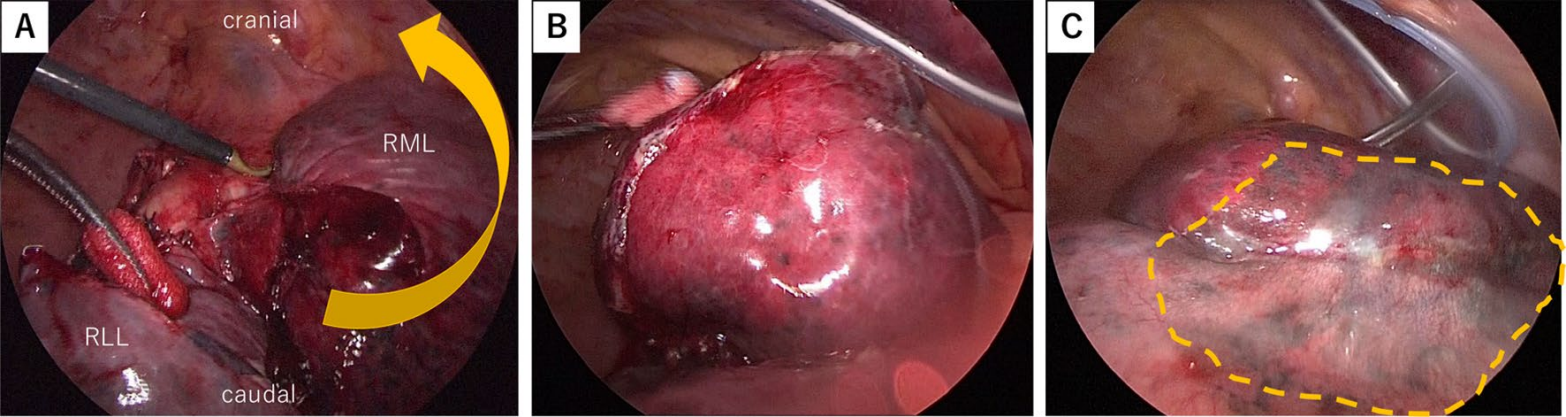
**A** Intrathoracic findings after right upper lobectomy with partial middle resection and S6 segmentectomy. **B** The middle lobe rotated easily when the lung was inflated. **C** The absorptive sheet, 0.15 mm thick (Neoveil: NV-M-015G, Gunze Ltd, Kyoto, Japan), was cut to approximately 50 × 33 mm. Each piece was soaked in 1.0 mL of liquid fibrinogen (Bolheal; KM Biologics Co, Kumamoto, Japan) and placed over the contiguous lobes in a bridging manner under lung inflation. Next, 1.0 mL of liquid thrombin was placed as drops onto the sheet, followed by fibrinogen and thrombin (1.0 mL of each), the latter of which were sprayed together to promote adherence. This fixation was performed in three locations for 5 min

**Fig. 3:**
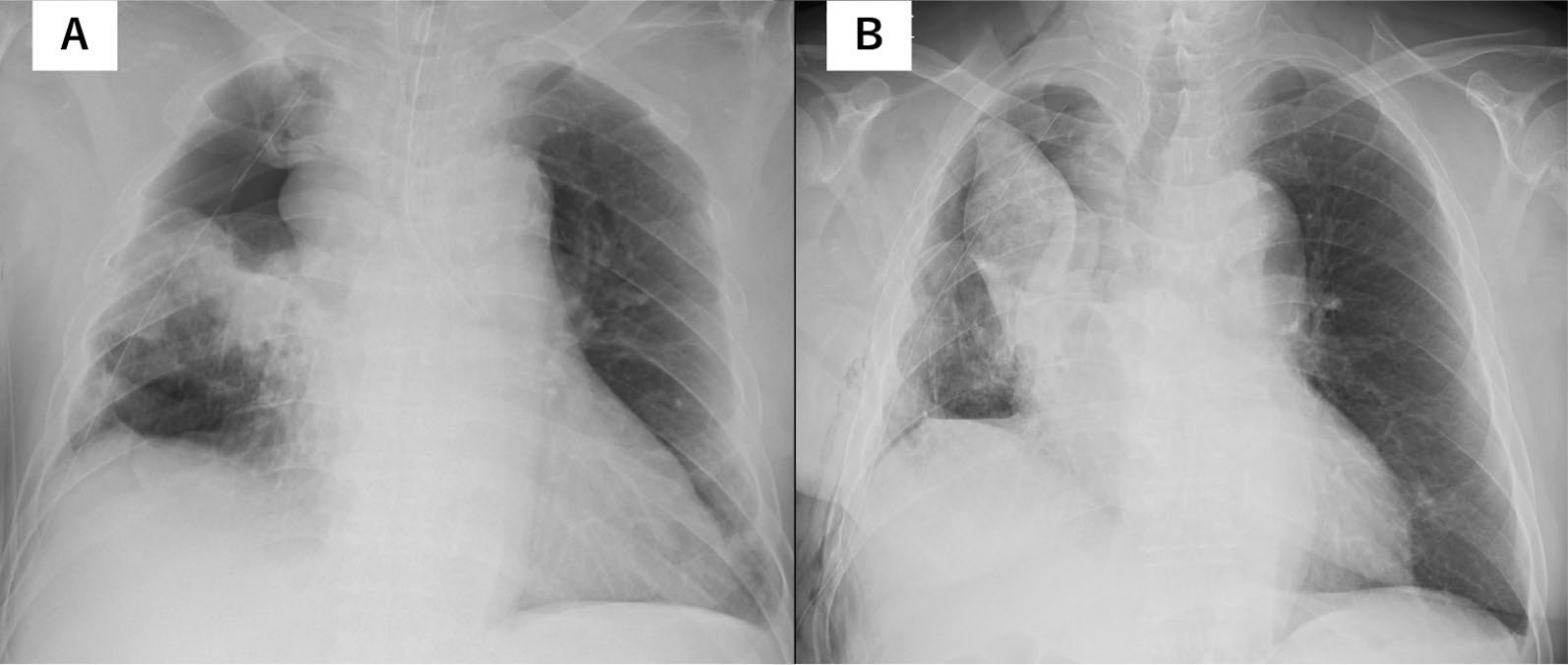
**A** Immediate postoperative chest radiograph. Lung expansion was confirmed. **B** Chest radiograph the day after surgery. Decreased middle lobe permeability and a cephaloparietal shift are observed

We report the case of a 77-year-old man who underwent a thoracoscopic right upper lobectomy with partial middle lobe resection and S6 segmentectomy for right upper lobe lung cancer (adenocarcinoma, pT2a (32 mm), N0M0) located at the confluence of the three right lung lobes and right lower lobe lung cancer (squamous cell carcinoma, pT1a (7 mm), N0M0) (Fig. 1Aa, Bb, and C). The middle and lower interlobular spaces were completely lobulated, and the pulmonary ligament was dissected at the time of lymph node dissection. Inversion of the middle lobe was observed during lung expansion before chest closure (Fig. 2A and B). A bridging structure with an absorptive sheet and fibrin glue was placed in the basal section of the middle lobe under lung expansion to prevent torsion (Fig. 2C). The operation was terminated after reconfirming lung expansion (Additional file 1). Postoperative chest radiographic and bronchoscopic findings confirmed the absence of torsion in the operating theater (Fig. 3A). On postoperative day 1, the patient was tachycardic. A chest radiograph revealed decreased lung field permeability (Fig. 3B). The patient underwent emergency surgery for suspected middle lobe torsion. On reoperation, dislocation of the bridging structure between the basal segments of the middle lobe was confirmed, and the middle lobe was observed to be deviated cephalad. In addition, pulmonary congestion in S4 due to pressure stenosis of V4 caused by the deviation of the middle lobe was observed, and middle lobe resection was performed (Additional file 2). The 1-year postoperative course was uneventful.

## Discussion

In the cohort study by Koike et al., approximately 90% of patients experienced the entire middle lobe migrating cephalad without rotation after right upper lobectomy; contrarily, approximately 10% of patients demonstrated the middle lobe rotating approximately 120° anticlockwise around the pulmonary hilum after right upper lobectomy, with more postoperative complications [5]. The present case represents 10% of the latter. In this case, the deviation of the residual middle lobe by different factors was considered a good division between the middle and lower lobes and the fact that the pulmonary ligament was dissected at the time of dissection. This was of double cancer case that required dissection. Fixation of the remaining lobe aids in preventing torsion and anticlockwise rotation. In the present case, despite using an absorptive sheet and a fibrin glue bridging structure in the basal segment of the middle lobe to prevent rotation, the structure was dislodged postoperatively, and the middle lobe rotated. This procedure was accompanied by an S6 segmentectomy with partial middle lobe resection, which resulted in a larger dead space in the thoracic cavity and increased mobility of the remaining middle lobe, excessive rotation, and torsion than normal upper lobe resection alone. The absorptive sheet and fibrin glue reinforcement method is simple and easy to use. This case suggested that the reinforcement method lacked sufficient strength to prevent middle lobe torsion, although this is the preferred practice in our department. If, as in this case, the middle lobe rotation is judged to be sufficiently strong when the lung is reinflated before chest closure, stronger fixation should be considered, including sutures, in combination with the use of collagen material, absorptive sheets, or fibrin glue [5].

## Conclusions

The absorptive sheet and fibrin glue reinforcement method lacked sufficient strength to prevent middle lobe torsion if the middle lobe rotation is thought to be sufficiently strong.

## Supplementary Information

**Additional file 1: Video 1.** Initial surgery.

**Additional file 2: Video 2.** At the time of reoperation.

## Data Availability

The datasets used and/or analyzed during the current study are available from the corresponding author upon reasonable request.

## Abbreviations

S6: Superior segment of the lower lobe
V4: Segmental pulmonary vein 4

## References

[1] Dai J, Xie D, Wang H, He W, Zhou Y, Hernández-Arenas LA (2016). Predictors of survival in lung torsion: a systematic review and pooled analysis. J Thorac Cardiovasc Surg.

[2] Cable DG, Deschamps C, Allen MS, Miller DL, Nichols FC, Trastek VF (2001). Lobar torsion after pulmonary resection: presentation and outcome. J Thorac Cardiovasc Surg.

[3] Fiorelli A, Scaramuzzi R, Costanzo S, Volpicelli A, Santini M (2015). Interlobar fixation using TachoSil(®): a novel technique. Transl Lung Cancer Res.

[4] Uramoto H, Takenoyama M, Hanagiri T (2010). Simple prophylactic fixation for lung torsion. Ann Thorac Surg.

[5] Koike S, Eguchi T, Matsuoka S, Takeda T, Miura K, Shimizu K (2022). Impact of counterclockwise rotation of the right middle lobe following right upper lobectomy. Interact Cardiovasc Thorac Surg.

